# The co-occurrence of both breast- and differentiated thyroid cancer: incidence, association and clinical implications for daily practice

**DOI:** 10.1186/s12885-022-10069-6

**Published:** 2022-09-26

**Authors:** Marceline W. Piek, Jan Paul de Boer, Frederieke van Duijnhoven, Jacqueline E. van der Wal, Menno Vriens, Rachel S. van Leeuwaarde, Iris M. C. van der Ploeg

**Affiliations:** 1 Department of Surgical Oncology, The Netherlands, Cancer Institute-Antoni Van Leeuwenhoek Hospital, Plesmanlaan 121, NL-1066 CX Amsterdam, The Netherlands; 2 Department of Medical Oncology, The Netherlands, Cancer Institute-Antoni Van Leeuwenhoek Hospital, Plesmanlaan 121, NL-1066 CX Amsterdam, The Netherlands; 3 Department of Pathology, The Netherlands Cancer Institute-Antoni Van Leeuwenhoek Hospital, Plesmanlaan 121, NL-1066 CX Amsterdam, The Netherlands; 4 Department of Endocrine Surgery, University Medical Centre of Utrecht, Heidelberglaan 100, 3584 CX Utrecht, The Netherlands; 5 Department of Endocrine Oncology, University Medical Centre of Utrecht, Heidelberglaan 100, 3584 CX Utrecht, The Netherlands

**Keywords:** Reast cancer, Thyroid cancer, Incidence, Association, Survival

## Abstract

**Background:**

Breast cancer (BC) and differentiated thyroid cancer (TC) are two common cancer types with the highest incidence in women. BC and TC can develop synchronous or metachronous and the occurrence of both is higher than expected by chance. This study aimed to examine the association between BC and TC in the Netherlands.

**Methods:**

This is a retrospective cohort study during the period of 1989–2020 retrieved from the Netherlands Cancer Registry (NCR). Patients diagnosed with BC-TC and BC alone as control group and TC-BC and TC alone as control group were included. The primary outcome was the standardized incidence ratio (SIR) of BC-TC and TC-BC. Secondary outcomes included data on the demographics, type of malignancy, treatment and overall survival (OS).

**Results:**

The incidence of TC among 318.002 women with BC (BC-TC) was 0.1% (423 patients) (SIR = 1.86 (95% CI: 1.40–2.32)) and the incidence of BC among 12,370 patients with TC (TC-BC) was 2.9% (355 patients) (SIR = 1.46 (95% CI: 1.09–1.83)). BC-TC patients were younger compared to the BC alone group at BC diagnosis (55 vs 60 years, *p* < 0.001). The age-adjusted odds ratio to develop TC was not significantly increased for patients who received chemotherapy and radiotherapy. Most TC cases were synchronous tumors after BC diagnosis (19%) with a TNM stage 1. Only 6% of the BC tumors after TC occurred synchronous with a TNM stage 1 in most cases. The OS of all groups was the most favorable in patients with both BC and TC compared to BC- and TC alone.

**Conclusion and relevance:**

The SIR of TC after BC diagnosis and BC after TC diagnosis was higher than predicted based on the rates of the general population. TC and BC as second primary tumors were diagnosed in an early stage and did not affect overall survival. Therefore, Dutch women who have been treated for BC or TC require no special surveillance for their thyroid- and breast gland.

## Background

Breast cancer (BC) is the most diagnosed cancer in women worldwide and the leading cause of cancer-related death in women [1]. Improvements in breast cancer screening, earlier detection and treatment options have resulted in increased long-term survival rates [2, 3]. Most women are diagnosed with early stage BC, with a 5-year survival rate of > 95% [4]. The relatively large group of breast cancer survivors may experience long-term treatment effects during follow-up. A considerable number of these long-term survivors may be at an increased risk of developing a second primary malignancy including thyroid cancer [2, 5]. Thyroid cancer is the most prevalent endocrine malignancy among women [6]. According to data from the Dutch national cancer registry, the incidence of thyroid cancer has increased in the Netherlands during the last decade with 906 new cases in 2021 [7, 8]. Well-differentiated papillary- and follicular thyroid cancers (TC) account for approximately 90% of cases and have an excellent 20-year disease specific survival rate of > 95% [9]. Given these favorable prognoses for both cancers, the risk of second primary malignancies is of particular concern [10].

Several articles have suggested that BC and thyroid cancer can develop synchronous or metachronous in patients and that the rate of occurrence is higher than expected by chance [2, 7, 11–13].

A large meta-analysis including patients from both Western- and non-Western countries demonstrated an increased risk of thyroid cancer as a secondary malignancy following BC [OR = 1.55; 95% confidence interval (CI), 1.44–1.67] and an increased risk of BC as a secondary malignancy following thyroid cancer (Odds ratio (OR) = 1.18; 95% CI, 1.09–1.26) [2]. In the Netherlands, the incidence of the occurrence of both diseases in the same patient and their possible correlation has not been extensively studied.

The hypotheses that may explain the higher incidence of one of these neoplasms following the other (BC-TC or TC-BC) is not clear [7, 11]. Common etiological factors, a genetic predisposition or a causal relation due to treatment-related factors, e.g. chemotherapy, radiotherapy and radioactive iodine therapy, might play a role in the association [2, 7, 11].

The aim of this study is to estimate the incidence of TC as a second primary cancer after BC and BC as a second primary cancer after TC in a large national population of the Netherlands. The second aim is to identify the clinicopathological characteristics, overall survival and possible associations between TC and BC. A greater understanding of the clinical relationship between BC and TC might help the post-treatment management of these patients.

## Methods

### Patient selection

The study population of this retrospective cohort study consists of patients with a first primary BC who subsequently developed TCr as a second primary malignancy and vice versa during the period of 1989–2020 in the Netherlands. The control groups consisted of patients with BC or TC without a second primary malignancy. The data was obtained from the Netherlands Cancer Registry (NCR) which collects data on all cancer patients diagnosed in the Netherlands, based on notification of newly diagnosed malignancies by the national automated pathological archive and of hospital discharge diagnoses. Information in the medical records on demographics, diagnosis, staging, and treatment is extracted routinely by specially trained NCR personnel. The survival status is updated annually using a computerized link with the national civil registry. For the present analysis, information on survival and second primary cancers was analyzed up to the end of 2020. The study population included patients ≥ 18 years with the diagnoses of TC or BC and the second primary malignancies for these patients. Only patients with differentiated thyroid cancer (TC) and/or invasive mammary carcinoma (BC) and/or ductal carcinoma in situ (DCIS) as a first primary- or second primary malignancy were selected for this study. Two cohorts were formed for further analyses: patients with BC followed by TC (BC-TC) versus BC alone as control group (first cohort) and patients with TC followed by BC (TC-BC) versus TC alone as control group (second cohort).

### BC and TC clinicopathological features

Variables of the included patients were reviewed and data on the type, features, treatment, and follow-up including the overall survival of BC and TC were collected and analyzed. Synchronous tumors refers to cases in which the second primary cancer was diagnosed within 6 months of the primary cancer. Metachronous tumors refers to tumors that were diagnosed more than 6 months after the diagnosis of the first primary cancer. The BC group was categorized in morphologically distinct groups based on immunohistochemistry. Estrogen (ER) and progesterone (PR) positivity were defined as tumor cell positivity of > 10%. Human epidermal growth factor receptor 2 (HER2) positivity was defined by immunohistochemistry (with FISH/SIHS when indicated). Clinical- and pathological Tumor-Node-Metastasis (TNM) staging was reported according to the 8th edition TNM classification by the American Joint Committee on Cancer (AJCC) for both BC and TC [14, 15]. Additionally, specific information about genetic counseling was collected for patients who were diagnosed and/or treated in the Antoni van Leeuwenhoek hospital (*n* = 31).

### Statistics

For the synchronous- and metachronous malignancies, standardized incidence ratios (SIR) were calculated as the ratio of the observed and expected number of cancer cases. In case of multiple metachronous cancers, only BC or TC as a second primary cancer was included in the analysis. The expected numbers were calculated by matching them with the incidence rates for the general population that were specified by age, gender, tumor type and year of diagnosis. Confidence intervals (95%) were calculated by assuming a Poisson distribution for the observed number of cases. A SIR of 1 means a similar incidence compared to the general population while a SIR of 10 indicates a tenfold higher incidence in the studied population. The analysis was performed in Stata version 17.0 and *p-*values were at 0.05 significance alpha levels.

Baseline variables including normally distributed numeric data were reported with the mean and standard deviation. Differences between both groups were analyzed using the two-tailed, independent sample t-test. Non-normally distributed numeric data were described with the median and interquartile range and group differences were analyzed using the Wilcoxon rank-sum test. For categorical data frequency and percentages were reported, and differences between groups were analyzed with the Fisher’s exact test or the Chi-squared test. Univariate logistic regression was performed to identify risk factors for the presence of TC and BC as second primary malignancies. The Kaplan–Meier method and an age-adjusted Cox proportional hazards model were used for the survival analysis. The OS was measured from the date of primary diagnosis (BC or TC) to the date of death from any cause, censoring patients who were still alive at the date of last contact. The OS was compared across the groups by using the log-rank test. A two-tailed *p* < 0.05 was considered statistically significant. These statistical analyses were conducted using R version 4.0.3.

## Results

### Patient selection

**Fig. 1:**
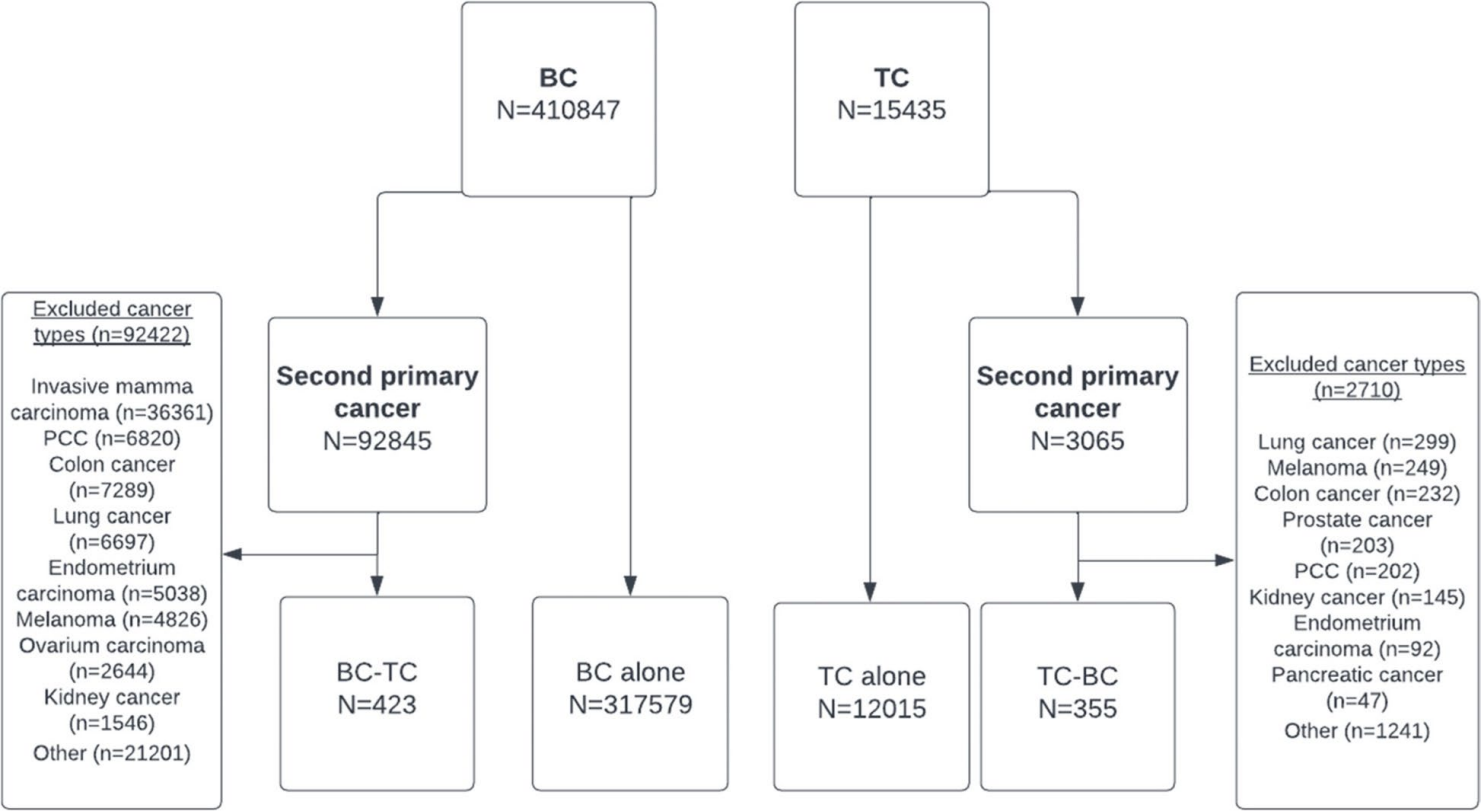
Flowchart of patient-selection

In total, 890 patients with medullary thyroid cancer and 911 patients with anaplastic thyroid cancer were excluded. Patients with a second primary malignancy other than BC or TC were also excluded. Figure 1 shows the patient-selection for this study. A total of 423 cases were identified in the BC-TC cohort with a control group of 317.579 patients with BC alone. The TC-BC cohort consisted of 355 patients with a control group of 12.015 patients with TC alone.

### Incidence of the BC-TC cohort and the TC-BC cohort

**Fig. 2:**
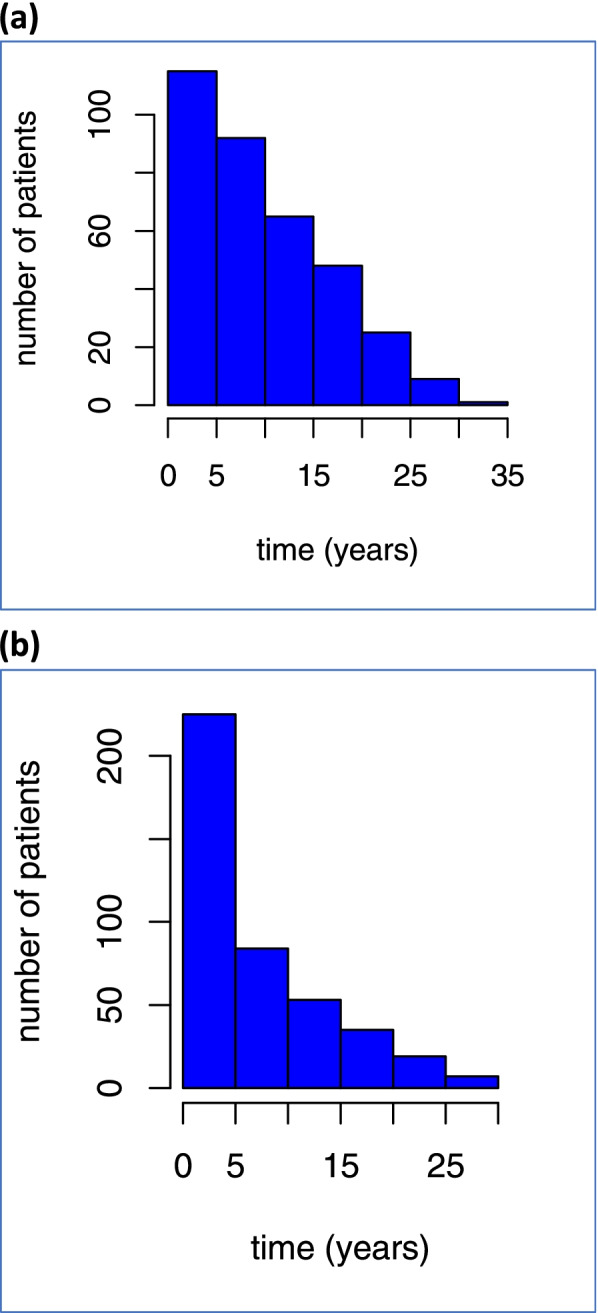
Histograms of the distrubition of new TC-BC (a) and BC-TC (b) patients

The incidence of TC among 318.002 women with BC was 0.1% (423 patients) (SIR = 1.86 (95% CI: 1.40–2.32)) and the incidence of BC among 12.370 patients with TC was 2.9% (355 patients) (SIR = 1.46 (95% CI: 1.09–1.83)). The median (IQR) interval between BC and TC was 4.2 (1.0–10.4) years and between TC and BC 8.2 (3.6–14.9) years. The highest incidence of TC as a second primary tumor following BC was observed during the first year of follow-up (26%) and in 80 patients (19%) the tumors were synchronous. Thirty-five patients (10%) with BC were detected during the first year of TC follow-up and in 21 patients (6%) the tumors were synchronous. In Fig. 2 the distribution of new patients with BC-TC and TC-BC is shown for the complete follow-up period.

### Features of BC patients followed by TC (BC-TC) and BC alone patients (1.st cohort)

  The clinicopathological features and treatment of the BC-TC group and BC alone group are presented in Table 1. Patients with BC followed by TC had a significantly younger age at the time of BC diagnosis (55 vs 60 years, *p* < 0.001) and consisted of only women. The majority of patients had a TNM stage I TC (51.1%) at the time of diagnosis. The median (IQR) tumor size of TC was 1.5 (0.8–2.5) cm and papillary thyroid carcinoma (PTC) was the most common subtype (78.3% of cases). Relatively more patients underwent chemotherapy (CT) in the BC-TC group compared to the BC alone group (37.4% vs 30.8% respectively, *p* < 0.001). Also, more patients underwent radiotherapy (RT) in the BC-TC group compared to the BC alone group (63.8% vs 58.8% respectively, *p* = 0.04). The age-adjusted odds ratio to develop TC was 0.91 (95% CI: 0.73–1.13, *p* = 0.4) for patients who received CT and 1.06 (95% CI: 0.87–1.30, *p* = 0.6) for patients who received RT. These non-sigificant associations suggest that age affects the indication for both CT, RT and the outcome TC.

**Table 1:** Clinicopathological features and treatment of breast cancer patients followed by thyroid cancer and breast cancer alone patients (1^st^cohort)

**Variables**	**BC‐TC (median (IQR)/%)**	**BC alone (median (IQR)/%)**	***p‐value***
No. of patients	423	317579	
**BC clinical characteristics**			
Age at BC diagnosis	55 (46-65)	60 (50-71)	< 0.001
Female	423 (100)	315808 (99.4)	0.2
Tumor size in mm	15 (10-25)	17 (11-25)	0.1
Tumor site			
Left	223 (52.7)	164071 (51.7)	1
Right	199 (47.0)	152907 (48.1)	
Unknown	1 (0.2)	601 (0.2)	
TNM stage			
0	38 (9.0)	29125 (9.2)	
I	154 (36.4)	112063 (35.3)	0.3
II	176 (41.6)	122649 (38.6)	
III	43 (10.2)	33816 (10.6)	
IV	9 (2.1)	16746 (5.3)	
M	0 (0.0)	1562 (0.5)	
Unknown	3 (0.7)	1618 (0.5)	
ER-positivity	201 (87.4^1^)	151727 (82.6^1^)	0.1
PR-positivity	154 (67.8^1^)	120837 (67.0^1^)	0.8
HER2-positivity	35 (17.5^1^)	23810 (14.8^1^)	0.5
BC surgery^2^	406 (96.0)	286592 (90.2)	<0.001
Breast-conserving	226 (53.3)	163501 (51.5)	
Ablative	168 (39.7)	13995 (35.9)	
Surgery NNO	12 (2.8)	8867 (2.8)	
BC radiation therapy	270 (63.8)	186779 (58.8)	0.04
BC chemotherapy	158 (37.4)	97895 (30.8)	<0.001
BC hormone therapy	191 (45.2)	140908 (44.4)	0.7
**TC clinical characteristics**			
Age at TC diagnosis	62 (52-72)		
Tumor size in mm	15 (8-25)		
Papillary thyroid cancer	331 (78.3)		
Follicular thyroid cancer	81 (19.1)		
Unknown histology	11 (2.6)		
Lymph node metastasis	98 (23.2)		
Distant metastasis	11 (2.6)		

^*1*^* Missings substracted *

^*2*^* Missing patients *

In subgroup analysis, two subsequent groups were formed consisting of Adolescents and Young Adults ages 18–39 years and patients > 39 years. The risk ratio to develop TC was not significantly increased for both subgroups of patients who received CT: 18–39 years: OR = 0.9, 95% CI: 0.7–1.3, *p* = 0.9 and > 39 years: OR = 0.9, 95% CI: 0.9–1.1, *p* = 0.8. The risk ratio to develop TC was also not significantly increased for patients aged 18–39 years who received RT (OR = 0.9, 95% CI: 0.7–1.3, *p* = 0.6). Patients who received RT > 39 years had a moderate increased risk for TC (OR = 1.12, 95% CI: 1.0–1.2, *p* = 0.02).

### Features of TC patients followed by BC (TC-BC) and TC alone patients (2.nd cohort)

The clinicopathological features and treatments of the TC-BC group and TC alone group are presented in Table 2. The median age at TC diagnosis in the TC-BC group was not significantly higher compared to the TC alone group (51 vs 50 years, *p* = 0.3). More patients with a TNM stage II and -III for TC were present in the TC-BC group compared to the TC alone group (25.0%, 15.5% vs 14.7% and 14.3%, *p* < 0.001). More patients in the TC-BC group underwent thyroid surgery compared to the TC alone group (*p* < 0.001). Most BC patients had a TNM stage 0, I or II at the time of BC diagnosis and invasive ductal carcinoma (81.1%) was the most common pathology result. The ER-, PR- and HER-2 positive rate was comparable in this TC-BC group and the BC alone group (84.7%, 68.4% and 14.6% vs. 82.6%, 67.0%, 14.8% respectively). The percentage of radioactive iodine (RAI) treatment was not significantly different between the TC-BC- and TC alone group.

**Table 2:** Clinicopathological features and treatment of thyroid cancer patients followed  breast cancer and thyroid cancer alone patients** (2**^nd^ cohort)

**Variables**	**TC‐BC (median (IQR)/%) **	**TC alone (median (IQR)/%)**	***p*‐value**
No. of patients	355	12015	
**TC clinical characteristics**			
Age at TC diagnosis	51 (42-61)	50 (38-64)	0.3
Female	353 (99.4)	8593 (71.5)	<0.001
Tumor size in mm	28 (18-39)	19 (9-34)	0.2
Tumor site			
Left	4 (1.1)	737 (6.1)	< 0.001
Right	5 (1.4)	1018 (8.5)	
Isthmus	2 (0.6)	103 (0.9)	
Ectopic	0	15 (0.1)	
Both sites	3 (0.8)	451 (3.7)	
Unknown	341 (96.1)	9623 (80.1)	
TNM stage			
I	186 (52.4)	7017 (58.4)	< 0.001
II	89 (25.0)	1769 (14.7)	< 0.001
III	55 (15.5)	1715 (14.3)	
IV	6 (1.7)	221 (1.8)	
IVa	10 (2.8)	667 (5.6)	
IVb	3 (0.8)	107 (0.9)	
IVc	2 (0.6)	312 (2.6)	
M	1 (0.3)	40 (0.3)	
Unknown	3 (0.8)	168 (1.4)	
TC surgery^2^	352 (99.2)	11351 (94.5)	< 0.001
Thyroidectomy	287 (80.8)	8954 (74.5)	
Hemithyroidectomy	65 (18.3)	2379 (19.8)	
Isthmusectomy	0	18 (0.1)	
TC radioactive iodine	252 (71.4)	8068 (67.1)	0.1
TC radiation therapy	6 (1.7)	280 (2.3)	0.7
**BC clinical characteristics **			
Age at BC diagnosis	61 (52-70)		
Tumor size in mm	15 (11-25)		
TNM stage	331 (78.3)		
0	53 (15.0)		
I	149 (42.0)		
II	99 (27.9)		
III	32 (9.0)		
IV	13 (3.7)		
M	1 (0.3)		
Unknown	8 (2.3)		
ER-positvity	211 (84.7^1^)		
PR-positivity	169 (68.4^1^)		
HER2-positivity	34 (14.6^1^)		

^*1*^* Missings substracted *

^*2*^* Missing patients*

### Overall survival of BC-TC and TC-BC group

The median (IQR) follow-up time in the BC-TC group was 25.2 (22.6–28.0) years and in the BC alone group 16.9 (16.8–17.0) years. The median (IQR) follow-up time in the TC-BC group was 25.4 (24.3–26.5) years and in the TC alone group 23.1 (22.8–23.4) years. Of 124.653 death events in de BC- and TC cohort, 124 occurred in the BC-TC group (29.3%) and 124.529 in the BC alone group (39.2%); 193.349 patients in total were censored without event.

Of 2903 deaths in the TC- and BC cohort, 93 occurred in the TC-BC group (26.2%) and 2810 in the TC alone group (23.4%); 9467 patients in total were censored without an event.

**Fig. 3:**
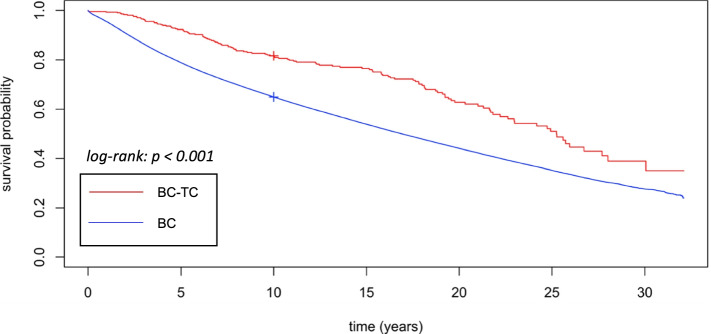
Kaplan Meier plot of overall survival of BC-TC patients and BC alone patients

**Fig. 4:**
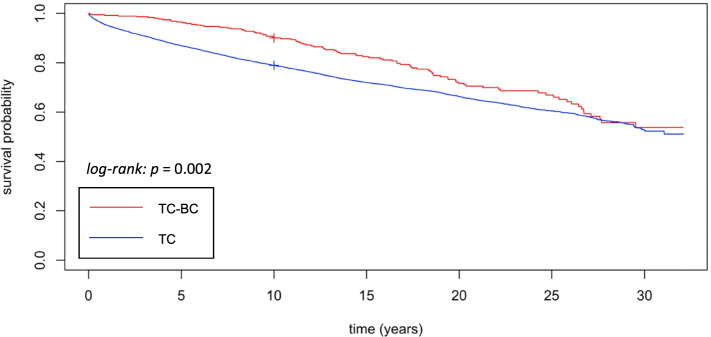
Kaplan Meier plot of overall survival of TC-BC group and TC alone group

The age-adjusted hazard ratio (HR) for OS was significantly higher in the BC-TC group compared to the BC alone group (HR = 1.4, 95% CI: 1.2–1.7, *p* < 0.001). The age-adjusted HR for OS in the TC-BC group compared to the TC alone group was also significantly increased (HR = 1.4, 95% CI: 1.1–1.7, *p* = 0.001). The survival curves are depicted in Fig. 3 and Fig. 4.

## Discussion

The SIR of TC after BC diagnosis (1.86) and the SIR of BC after TC diagnosis (1.46) was higher than predicted based on the rates of the general population. BC-TC patients were younger compared to the BC alone group (55 vs 60 years) at BC diagnosis and consisted of women only. Papillary thyroid carcinoma (PTC) and invasive ductal carcinoma respectively were the most common pathology results for second primary malignancies in the BC-TC and TC-BC group. Most TC diagnoses were depicted in the first year after BC treatment and were mostly TNM stage I. When comparing the studied cohorts, OS was less favorable in the BC alone group compared to the BC-TC group and in the TC alone group compared to the TC-BC group.

### Association between BC and TC

The possible association between TC and BC has been suggested before. The higher incidence of thyroid cancer in BC patients was confirmed by previous studies [2, 12, 13]. Joseph et al. performed a meta-analysis of 18 studies and demonstrated a significantly increased risk of thyroid cancer after a primary diagnosis of BC (BC-TC) ((SIR) = 1.59, 95% CI: 1.28–1.99, *p* < 0.01) [12]. This SIR is slightly lower compared to our study (SIR = 1.86; 95% CI: 1.40–2.32, *p* < 0.01). Another large meta-analysis showed an odds ratio of developing thyroid cancer as a secondary malignancy following BC of 1.55 (95% CI: 1.44, 1.67) [2]. A previous study that was performed in the Netherlands included 9919 BC patients and observed a lower incidence of thyroid cancer among BC patients compared to our study (0.02% vs 0.1% respectively) [13]. This might be explained by the higher prevalence of thyroid cancer in the general population of the Netherlands in the last decade (340 new thyroid cancer patients in 1990 compared to 800 new thyroid cancer patients in 2020) [8].

The incidence of thyroid cancer among 13,978 patients with BC in a Chinese cohort was 1.8% and the associated SIR was 4.48 compared to the general population [11]. This higher SIR might be driven by overdiagnosis of thyroid cancer in China [16]. Similarly, two meta-analyses showed a marginally increased risk of developing breast cancer as a second primary malignancy of thyroid cancer (SIR = 1.24, [95% CI:1.16–1.33], SIR = 1.25 [95% CI: 1.17–1.32]) compared to the general risk of developing a primary malignancy following thyroid cancer [12, 17]. This number is also moderately lower compared to our study results.

### Factors influencing BC and TC

The clinicopathological features of BC-TC and TC-BC patients were studied before. Studies show that the increased risk of BC among thyroid cancer patients was higher in younger women under the age of 50 at the time of diagnosis [18–21]. However, the age of TC-BC patients in our cohort was comparable to the TC alone group. Li et al. also showed a significantly younger median age at the time of BC diagnosis in BC followed by thyroid cancer patients (54 vs 59, *p* < 0.001) [22]. In our cohort, BC-TC patients were also significantly younger compared to the BC alone group (55 vs 60, *p* < 0.001). This finding might also explain the relatively better survival in the BC-TC group during the first ± 18 years compared to the BC alone group (Fig. 3).

Despite several studies demonstrating an association between breast- and thyroid cancer, the mechanisms in which they are related remains elusive. Hypotheses focus on common etiologies such as hormonal, genetic, environmental, and therapeutic factors [2]. Another non-disease related factor includes the stringent cancer screening of these patients at a younger age which can result in more incidental findings [23]. This can cause a lower stage of BC or TC at the time of diagnosis and probably a higher incidence. This study showed a relatively higher number of patients with DCIS in the TC-BC group compared to the BC alone group (15.0% vs. 9.2%). This finding is consistent with the study of Canchola et al. in which an increased incidence of DCIS following thyroid cancer was found compared to invasive breast cancer [24]. The tumor size of TC in the BC-TC group was smaller (Table 1) compared to the TC tumor size in the TC alone group (Table 2) (1.5 cm vs. 1.9 cm, *p* = 0.03) which is consistent with previous results [25]. It has also been demonstrated that the risk for thyroid cancer was significantly higher during the first 3 years of follow-up in BC patients compared to the general population (SIR 1.22, 95% CI [1.14, 1.31]) [22]. The study of An et al. confirmed this with a higher incidence of second primary thyroid cancer the first 5 years after BC diagnosis [26]. These findings are consistent with our results and may reflect an increase in imaging surveillance in patients when diagnosed with BC. Follow-up of breast cancer treatment in the Netherlands aims for an early detection of locoregional breast cancer recurrences and metastastic disease [27]. Fluorodeoxyglucose-positron emission tomography/computed tomography also called a FDG-PET/CT scan was found to be useful in the first 5 years of follow-up of BC patients with BRCA1/2 mutations [28]. In 2% of PET/CT examinations, FDG-thyroid incidentaloma are detected with a thyroid cancer prevalence of 35–40% [29]. These incidental findings may explain the increased TC incidence during the first 5 years of BC follow-up in our study.

Other theories focus on hormonal factors because the breast and thyroid are glands regulated by the hypothalamic-pituitary axis. Breast-  and thyroid cancer are both predominantly hormone-related cancers with a specific carcinogenic mechanism [30]. Both breast- and thyroid cancer demonstrate a gender disparity favoring women in this study. Both ER and PR have potential effects on thyroid cell- and breast cell proliferation [31, 32]. ER is a potent growth factor for malignant thyroid cells and it was demonstrated that the levels of ER were significantly higher in thyroid cancer compared to normal thyroid tissue [32, 33]. Both ER and PR are thought to have functional roles in MCF-7 breast cell proliferation [31]. Previous studies have shown that the expression of ER receptors and PR receptors were significantly higher in BC patients with co-existing thyroid cancer, compared to those without [26, 34, 35]. However, the results of this study show a similar ER- and PR receptor rate in the TC-BC cohort compared to the BC alone group. The role of thyroid hormone in the development of breast cancer remains uncertain [36].

Other potential etiological factors are related to the treatment for the primary malignancy. The use of radioactive iodine (RAI) treatment has been studied in relation to the higher risk for BC in thyroid cancer patients. RAI treatment did not seem to increase the risk of BC in previous studies and our study observations are consistent with these previous results [37–40].

Marcheselli et al. showed that chemotherapy and radiotherapy in BC patients were related to an increased risk of developing any second cancer, whereas hormonal therapy (HT) had a protective effect [41]. A recent prospective study showed that hormone replacement therapy (HRT) with ER alone was associated with an increased risk of thyroid cancer (HR 1.67, 95% CI 1.08–2.59) [42]. However, HT had no protective effect for the development of TC in our study. Radiotherapy is the most extensively researched treatment-related factor in the association between BC and the risk for thyroid cancer. Some studies have shown that radiation can cause thyroid disorders such as hypothyroidism, Graves’ disease and thyroid cancer [43–47]. Another study could not confirm this finding [48]. The study of Sun et al. showed that younger patients with BC exhibited a significantly higher risk of thyroid cancer than those in the comparison control cohort, regardless of whether they received RT [49]. The current study shows that patients > 39 years who received RT for BC had a moderate increased risk for developing TC. In the study of Lin et al., CT for BC did not significantly increase HRs for the risk of thyroid cancer (adjusted HR = 1.02, 95% CI 0.62–1.66) [50]. Our age-adjusted odds ratio also showed no significant association between chemotherapy and an increased risk for TC. The associations remained insignificant after stratification by age groups (18–39 years and > 39 years).

Genetic factors have also been suggested as a possible explanation for the association between BC and thyroid cancer. The Cowden syndrome is currently the only tumor syndrome known that increases the risk of developing both breast and thyroid cancer in the same individual [2]. Mutation in the tumor suppressor PTEN gene is the most common cause of Cowden syndrome [2, 51]. In our study, a small number of patients (N = 31) underwent genetic counseling in the Antoni van Leeuwenhoek hospital and therefore had the results available. One of these patients had Cowden syndrome (CS) and the associated mutation in the tumor suppressor gene PTEN. In a previous study, the propensity for breast and thyroid cancer as a second primary malignancy was higher in individuals with PTEN mutations (SIR = 8.92 for breast cancer and SIR = 5.83 for thyroid cancer) [2, 51]. The study of Bakos et al. could not identify mutations in the PTEN gene in patients with synchronous breast and thyroid cancer [52]. They did report an increased burden of single nucleotide polymorphisms (SNPs) in patients with thyroid cancer and BC compared to patients with BC alone. If more genetic sequencing is brought into clinical practice, more variants and mutations might be identified.

### Strengths and limitations

The main strength of this study is that it represents a comprehensive examination of the incidence and association of BC-TC and TC-BC for a large population in the Netherlands during a long follow-up period. Limitations of this study are related to the data source. For example, detailed information about genetic counseling was lacking for most patients outside of the Antoni van Leeuwenhoek hospital. Due to the retrospective nature of this study, missing information could not be verified. The cancer-specific survival was also not available through the NCR.

### Clinical implications

The SIR of TC after BC diagnosis (SIR 1.86) and BC after TC diagnosis (SIR 1.46) was higher than predicted based on the rates of the general population in the Netherlands. Treatment related factors such as chemotherapy and radiotherapy were not associated with developing BC followed by TC after adjusting for age. TC as second primary tumor was diagnosed in an early stage and did not compromise survival. Therefore, special surveillance of the thyroid gland in BC survivors is not advisable. All women aged 50–75 years in the Netherlands are enrolled in the national BC screening program. Earlier or more frequent surveillance of the breast gland in TC survivors is not recommended.

## Data Availability

All data generated or analysed during this study are included in this published article.

## Abbreviations

BC: Breast cancer
DCIS: Ductal carcinoma in situ
TC: Differentiated thyroid cancer
SIR: Standardized incidence ratio
vs: Versus
OS: Overall survival
OR: Odds ratio
ER: Estrogen
PR: Progesterone
HER2: Human epidermal growth factor receptor 2
TNM: Tumor nodes metastases
IQR: Interquartile range
CT: Chemotherapy
RT: Radiotherapy
HT: Hormonal therapy
HR: Hazard ratio
RAI: Radioactive iodine
FDG-PET/CT: Fluorodeoxyglucose-positron emission tomography/computed tomography
